# Changes of macular blood flow and structure in acute primary angle closure glaucoma

**DOI:** 10.1007/s10792-022-02399-y

**Published:** 2022-07-04

**Authors:** Rui Wang, Jin Yang, Liukun Shi, Yue Qu, Dan Xu, Yufeng Liu, Xuan Li

**Affiliations:** Tianjin Key Lab of Ophthalmology and Visual Science, Tianjin Eye Hospital, Tianjin Eye Institute, No.4.Gansu Road, He-ping District, Tianjin, China

**Keywords:** Acute primary angle closure glaucoma, Foveal avascular zone, Vessel density

## Abstract

**Purpose:**

We assessed the relationship between acute primary angle closure glaucoma (APACG) severity and macular microcirculation, as well as the diagnostic ability of blood flow and macular structural parameters on optical coherence tomography angiography (OCTA) for APACG.

**Methods:**

APACG patients were assigned to mild, moderate, and severe groups in this cross-sectional study. Age-matched primary angle closure suspect (PACS) and healthy control groups were also recruited. The vessel density (VD) and foveal avascular zone (FAZ) in each macular superficial area were measured using OCTA. The retinal nerve fiber layer thickness (RNFLT) and ganglion cell complex thickness (GCCT) of the corresponding regions were measured using OCT.

**Results:**

All parameters in the control, PACS, and mild APACG groups differed significantly from those in the moderate and severe APACG groups (all *P* < 0.05). VD and RNFLT showed high and moderate diagnostic ability, respectively, to distinguish moderate APACG from PACS, with significant differences (*P* < 0.05) in areas under the receiver operating characteristic curve (AUCs) for VD and RNFLT in six macular areas. The diagnostic abilities of VD and RNFLT for distinguishing severe APACG from PACS were increased, with significant differences in the AUCs for VD and RNFLT in five macular areas (*P* < 0.05). All macular VDs and GCCTs were similar among the three APACG groups (*P* > 0.05).

**Conclusions:**

Damage to the VD and FAZ in the macula increased with APACG severity. VD in the macular superficial layer showed a higher diagnostic ability than RNFLT, which was equivalent to that of GCCT.

## Introduction

Glaucoma is defined as a multifactorial optic neuropathy. Increasing evidence shows that vascular factors play an important role in the pathogenesis of this disease [1, 2]. However, the study of the blood flow in glaucoma remains challenging because of the limitations of imaging methods [3, 4]. A large-sample epidemiological survey revealed that the incidence of primary angle closure glaucoma (PACG) accounts for nearly one-third of all glaucoma patients worldwide [5] and is higher in Asians than in other populations, with more than 80% of all PACG cases occurring in Asia [6]. Acute transient intraocular pressure (IOP) elevation and its duration during an attack of acute PACG (APACG) not only have important effects on optic disc morphology [7], retinal nerve fiber layer thickness (RNFLT) [8], but also have a marked impact on retinal microcirculation [9, 10]. In a study investigating long-term visual outcomes in Asians after acute primary angle closure (APAC) event for several years, it was found that almost half of subjects suffered from glaucomatous optic neuropathy (GON) and a third of patients had severe cup-to-disc ratio changes [11]. In the past, it was found that the diameter of retinal blood vessels in PACG patients was significantly smaller than that in primary angle closure suspect patients in normal control group by digital imaging measurement, which may result in changes in retinal blood vessel circulation and subsequent GON [12].

Optical coherence tomography angiography (OCTA) is a noninvasive imaging technique that allows reproducible quantitative assessment of retinal and choroidal vascular systems [13], which are divided into the superficial, deep, and outer retina, and the choroidal vascular layer. OCTA was used to detect the filling of retinal capillaries in rats with acute IOP elevation. When IOP increased to 100 mmHg, the retinal blood flow decreased linearly [14]. Although there is autonomic regulation of retinal blood vessels to a certain extent [15–17], when IOP rises to a certain level, it will cause retinal blood circulation to stop and temporary central retinal artery occlusion. After IOP is controlled, recanalization of the partial microcirculation systems may occur, but irreversible damage exists in retinal blood vessels in special parts, such as radial peripapillary capillaries [18] and macular microcirculation system [10]. Even if IOP is controlled stably after many years, irreversible damage can be detected, thus participating in the pathogenesis of glaucoma. Therefore, the sharp increase of IOP can not only bring temporary effects on retinal blood flow, but also result in long-term and extensive damage. Therefore, studying APACG-affected eyes with a history of acute attack may provide a unique opportunity to study the association between VD and glaucoma. Moreover, structural damage in glaucomatous eyes often precedes detectable visual field defects [19, 20]. However, few studies are available regarding the relationship between VD damage and structural damage, and their changes during disease progression.

Retinal ganglion cells (RGCs) are important damage markers in glaucoma, and are mainly present in the superficial retina [21]. Approximately half of the RGCs are located in the central fovea (4.5 mm). The superficial capillary network is the main source of nutrients for RGCs [22]. After APAC onset, the changes of microcirculation around RGCs may be related to the secondary degeneration of RGCs [23]. The foveal avascular zone (FAZ) is a nearly circular, noncapillary region located in the central fovea of the macula that is surrounded by interconnected capillary beds [24] and that can be detected by OCTA. Histologically, the FAZ boundary is formed by a single-layer capillary arcade located inside the RGC layer.

This study aimed to assess the relationship between the severity of APACG and macular microcirculation, as well as the diagnostic ability of blood flow and macular structural parameters, determined using OCTA, for APACG. To this end, we investigated changes in the macular microcirculation in eyes with different APACG severity.

## Patients and methods

### Subjects

Patients with APACG who were hospitalized at the Tianjin Eye Hospital between January and April 2019 were consecutively enrolled. The study was approved by the ethics committee of Tianjin Eye Hospital (registration number of Chinese Clinical Trial Registry: ChiCTR1900021086; review approval of the Ethics Committee of Tianjin Eye Hospital: TJYYLL-2018-05) and followed the tenets of the Declaration of Helsinki for research involving human subjects. All participants provided written informed consent.

All subjects were between 50 and 80 years of age. We recruited patients with APACG as well as age-matched patients with primary angle closure suspect (PACS) and individuals with healthy eyes.

The inclusion criteria for APACG were as follows: (1) Patients with a previous history of APAC, within 6 weeks after onset, whose IOP was controlled within the normal range after drug or laser treatment. APAC was defined based on the following criteria: (i) at least two of the following symptoms: eyeball pain, nausea, vomiting, or both; history of intermittent visual blurring; and (ii) IOP exceeding 21 mmHg (based on Goldmann applanation tonometry [GAT]), with at least three of the following signs: conjunctival congestion, corneal edema, moderate pupil dilation, and shallow anterior chamber; and (iii) gonioscopy revealing angle closure [25, 26]. (2) Patients with glaucoma-related optic nerve or visual field defects.

The inclusion criteria for individuals with PACS were as follows: (1) Static angle closure > 180°; and (2) IOP < 21 mm Hg without glaucomatous optic neuropathy [27].

The exclusion criteria, for all subjects, were as follows: patients with high myopia or high hypermetropia (mean diopter ≤  ± 6D); patients with a history of any retinal diseases, non-glaucomatous optic neuropathy, uveitis, ocular trauma, or ocular diseases that seriously affected the turbidity of the refracting media of the eyeball; patients with a history of ocular surgery; and patients with moderate and above diabetes or hypertension [28].

The age-matched healthy control group consisted of 33 eyes, PACS group consisted of 40 eyes and APACG groups consisted of 109 eyes. APACG groups were further divided into three subgroups according to severity. Using the grading method of the Glaucoma Staging System [29], APACG subjects at stage 1 were categorized into the mild group (35 eyes), those at stage 2 into the moderate group (41 eyes), and those at stage 3–5 into the severe group (33 eyes).

All participants underwent complete ophthalmological examinations 6 weeks after the onset of APAC, including best-corrected visual acuity and spherical equivalent (SE) by refractive error examination, slit-lamp examination, gonioscopy, and ophthalmoscopy, as well as IOP measurement by GAT during OCTA evaluation. Lenstar LS900 (version V4.2.1; Haag-Streit, Switzerland) was used to measure axial length (AL), anterior chamber depth (ACD), and lens thickness (LT). Mean deviation (MD) values were measured for all subjects using standard automated perimetry (Humphrey Field Analyzer, 30-2; Carl Zeiss Meditec, CA, USA).

### OCTA examination

**Fig. 1:**
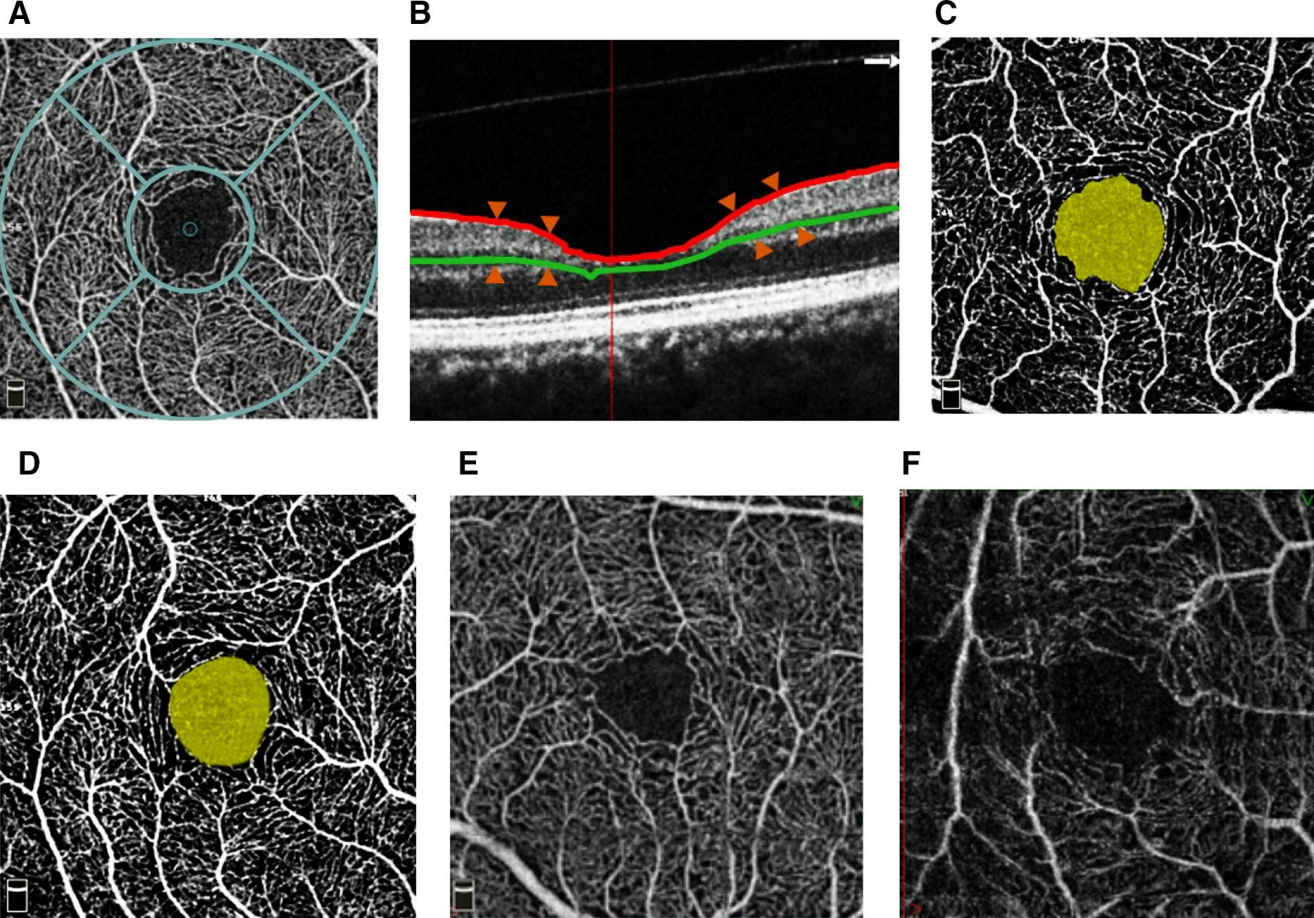
Image captured by OCTA. **a** The zonal image of the VD in the macula was divided into the macular central fovea and four paracentral foveae (temporal, superior, nasal, and inferior). The paracentral foveae were located in two concentric circles of 1 and 2.5 mm diameter. **b** The measurement range of VD, including the thickness of the inner limiting membrane, nerve fiber layer, ganglion cell layer, and inner plexiform layer. **c** In the APACG group, the capillaries around FAZ were sparse and anfractuous in trend, and the arch ring was enlarged with irregular morphology. **d** In the normal control group, the capillaries around FAZ were arachnoid, and the arch ring was complete and regular in morphology. **e** At the image capture, the image was obviously shifted due to factors, such as blinking, and was not used. **f** The scanning signal intensity was less than 60. The image had a poor effect, and therefore it was not used

After admission, all participants immediately underwent an OCTA examination, using RTVue XR (software version 2016.1.0.26; Optovue Inc., Fremont, CA, USA) instruments, by two specially trained physicians. They measured the VD of the superficial retina (including the inner limiting membrane, nerve fiber layer, ganglion cell layer, and inner plexiform layer) within 3 × 3 mm^2^ centered on the macula. The measurement area was divided into the macular fovea (F) and parafovea (PF), which were further divided into the superior hemiretina (SH), inferior hemiretina (IH), temporal (T), superior (S), nasal (N), and inferior (I) sectors, while the FAZ area was automatically measured. A light source with a wavelength of 840 nm was used for the OCTA examination, and the scanning speed was 70 kHz. Scanning was performed once in the horizontal and vertical directions. Patients were instructed to stare at the fixation point without blinking. A system with eyeball tracking and motion correction function was initiated, and the final image quality was checked by the operator. The vessel connection needed to be intact with no motion artifacts and vitreous floaters, and the signal strength index (SSI) needed to be greater than 60%; otherwise, the data were not used. The RNFLT in the corresponding region was obtained using the same protocol as that used for VD measurement. The thickness of the ganglion cell complex (GCCT) (i.e., the thickness of the nerve fiber layer, ganglion cell layer, and inner plexiform layer) was measured using the GCCT protocol of the system, and the average value of each subarea was automatically calculated (Fig. 1).

### Statistical analysis

The numeric variables were confirmed to be normally distributed using the Shapiro‒Wilks *W* test, calculated as mean and standard deviation, and categorical variables, such as the sex ratio, represented by the composition ratio. Using SPSS v18.0 statistical software (SPSS Inc., Chicago, IL), one-way analysis of variance was used to compare the differences in basic clinical characteristics, VD, and FAZ in each macular area among the five groups. If the variances of each group were homogeneous, Bonferroni-corrected post-hoc *t*-tests were used for each group comparison. Alternatively, Dunnett’s T3-corrected post-hoc *t*-tests were used for each group comparison. The chi-square test was used to determine the constituent sex ratio and the ratio of systemic diseases in the five groups. Using R 4.1.1 software, spearman correlation analysis was used for the correlation between visual field values and VD, FAZ, and R package GGplot2 was used to draw scatter graphs. MedCalc v18.2.1 (MedCalc Statistical Software, Mariakerke, Belgium) was used for areas under the receiver operating characteristic curve (AUC) analysis to assess the diagnostic abilities of VD, RNFLT, and GCCT in the macula for APACG. Statistical significance was set at *P* < 0.05.

## Results

The demographics and clinical characteristics of the experimental subjects are shown in Table 1. The differences in the constituent ratio of sex and systemic diseases, age, IOP, SE, and SSI among the five groups were not statistically significant (*P* > 0.05). As expected, compared to the healthy control group and the PACS group, the three groups of APACG had significantly more glaucoma medications, shorter AL, shallower ACD, thicker LT, and larger MD values (*P* < 0.05). However, there were no statistically significant differences among the mild, moderate, and severe APACG groups in the AL, ACD, LT, number of acute attacks, and the highest IOP during an acute attack (*P* > 0.05). However, the MD values, C/D ratio, and the number of glaucoma medications among the mild, moderate, and severe groups were significantly different (*P* < 0.05).

**Table 1:** Demographics and clinical characteristics of study subjects

Mean ± SD	healthy control	PACS	Mild	Moderate	Severe	*P* Value
Case number	33	40	35	41	33	–
Sex^a^, *n* (%)						
Male	16 (48.48)	51 (46.79)	17 (48.57)	19 (46.34)	15 (45.45)	0.93
Female	17 (51.52)	58 (53.21)	18 (51.43)	22 (53.66)	18 (54.55)	
Age^b^ (years)	63.84 ± 9.35	65.47 ± 9.38	66.98 ± 10.93	64.32 ± 7.58	64.84 ± 8.84	0.56
Hypertension^a^, (yes: no)	10: 23	14: 26	15: 20	16: 25	11: 22	0.84
Diabetes^a^, (yes: no)	9: 24	11: 29	10: 25	13: 28	8: 25	0.97
Ischemic encephalopathy^a^, (yes: no)	3: 30	2: 38	5: 30	1: 40	2: 31	0.33
Number of glaucoma medications^b^	0.00 ± 0.00	0.20 ± 0.41	1.80 ± 0.96	2.61 ± 0.86	3.42 ± 1.09	≤ 0.01*
Number of acute attacks^b^	0.00 ± 0.00	0.00 ± 0.00	1.94 ± 0.94	2.76 ± 1.09	3.00 ± 1.35	≤ 0.01*
The highest IOP during acute attack^b^ (mm Hg)	15.69 ± 3.11	16.00 ± 3.20	47.64 ± 8.71	51 ± 9.62	51.97 ± 6.65	≤ 0.01*
IOP during examination^b^ (mm Hg)	17.15 ± 2.95	18.89 ± 7.16	17.89 ± 4.55	19.48 ± 9.77	15.88 ± 4.97	0.12
SE^b^ (D)	-0.31 ± 1.96	0.48 ± 2.29	0.30 ± 2.23	0.20 ± 3.12	0.26 ± 2.38	0.54
AL^b^ (mm)	23.56 ± 1.99	22.77 ± 1.00	22.11 ± 0.99	22.51 ± 0.97	22.39 ± 1.03	≤ 0.01*
ACD^b^ (mm)	2.54 ± 0.38	2.10 ± 0.20	1.99 ± 0.20	2.09 ± 0.23	2.01 ± 0.13	≤ 0.01*
LTf^b^ (mm)	4.57 ± 0.45	4.68 ± 0.54	4.92 ± 0.38	4.89 ± 0.34	4.90 ± 0.36	0.04*
MD^b^ (dB)	0.25 ± 0.65	0.14 ± 0.60	-3.80 ± 1.44	-9.13 ± 1.70	-17.16 ± 4.11	≤ 0.01*
C/D^b^ ratio	0.25 ± 0.19	0.26 ± 0.19	0.40 ± 0.21	0.65 ± 0.09	0.77 ± 0.13	≤ 0.01*
SSI^b^ (%)	73.42 ± 7.22	72.17 ± 7.01	72.40 ± 7.21	74.49 ± 6.60	71.21 ± 7.25	0.28

*PACS* primary angle closure suspect; *IOP* intraocular pressure; *SE* spherical equivalent; *AL* axial length; *ACD* anterior chamber depth; *LT* lens thickness; *C/D* cup/disk; *MD* mean deviation; *SSI* signal strength index

*P < 0.05, showing that the difference was statistically significant

^a^Statistical analyses were performed using chi-square test

^b^Statistical analyses were performed using one-way ANOVA test

The differences in all parameters were found to be statistically significant among the five groups (*P* < 0.05). Further pairwise comparisons were performed for each group. Except for F-VD and FAZ, differences in other parameters were not significantly different among the healthy control, PACS, and mild APACG groups (*P* > 0.05). Compared with the healthy control and PACS groups, the F-VD in the mild group significantly decreased and FAZ significantly increased, and the difference was statistically significant (*P* < 0.05). Comparing the healthy control, PACS, and mild groups with the moderate and severe groups, differences in all parameters were statistically significant (*P* < 0.05), indicating that with the exacerbation of APACG, VD gradually decreased and FAZ gradually increased. Except for IH-VD, T-VD, and I-VD, differences in parameters were statistically significant between the moderate and severe groups (*P* < 0.05), which indicated that with the exacerbation of APACG, the VD decreased and FAZ increased (Table 2).

**Table 2:** Effects of different degrees of APACG on macular VD and FAZ

	Healthy control	PACS	Mild	Moderate	Severe	*P*
F-VD (%)	26.28 ± 4.28	24.86 ± 4.58	23.44 ± 4.55	21.19 ± 3.96	21.24 ± 4.03	≤ 0.01*
PF-VD (%)	53.24 ± 2.61	52.65 ± 3.18	52.52 ± 3.47	48.94 ± 3.26	47.44 ± 4.19	≤ 0.01*
SH-VD (%)	53.76 ± 2.32	52.71 ± 3.22	52.82 ± 3.61	49.41 ± 3.73	47.85 ± 4.04	≤ 0.01*
IH-VD (%)	52.71 ± 3.14	52.31 ± 3.32	52.22 ± 3.40	48.47 ± 4.07	47.02 ± 4.77	≤ 0.01*
T-VD (%)	51.80 ± 2.93	51.32 ± 3.27	51.19 ± 4.27	49.08 ± 3.00	47.86 ± 3.11	≤ 0.01*
S-VD (%)	54.58 ± 3.57	53.480 ± 3.48	53.300 ± 4.22	48.70 ± 3.29	46.96 ± 3.93	≤ 0.0*
N-VD (%)	52.43 ± 4.86	52.10 ± 3.22	52.78 ± 4.84	49.83 ± 3.96	46.62 ± 5.51	≤ 0.01*
I-VD (%)	54.14 ± 4.28	53.12 ± 4.02	52.82 ± 5.06	48.20 ± 5.85	48.35 ± 4.57	≤ 0.01*
FAZ (mm^2^)	0.29 ± 0.04	0.36 ± 0.06	0.38 ± 0.03	0.44 ± 0.05	0.45 ± 0.05	≤ 0.01*

*APACG* acute primary angle closure glaucoma; *PACS* primary angle closure suspect; *VD* vessel density; *FAZ* foveal avascular zone; *F* fovea; *PF* parafovea; *SH* superior hemiretina; *IH* inferior hemiretina; *T* temporal; *S* superior; *N* nasal; *I* inferior

*P < 0.05, showing that the difference was statistically significant

**Fig. 2:**
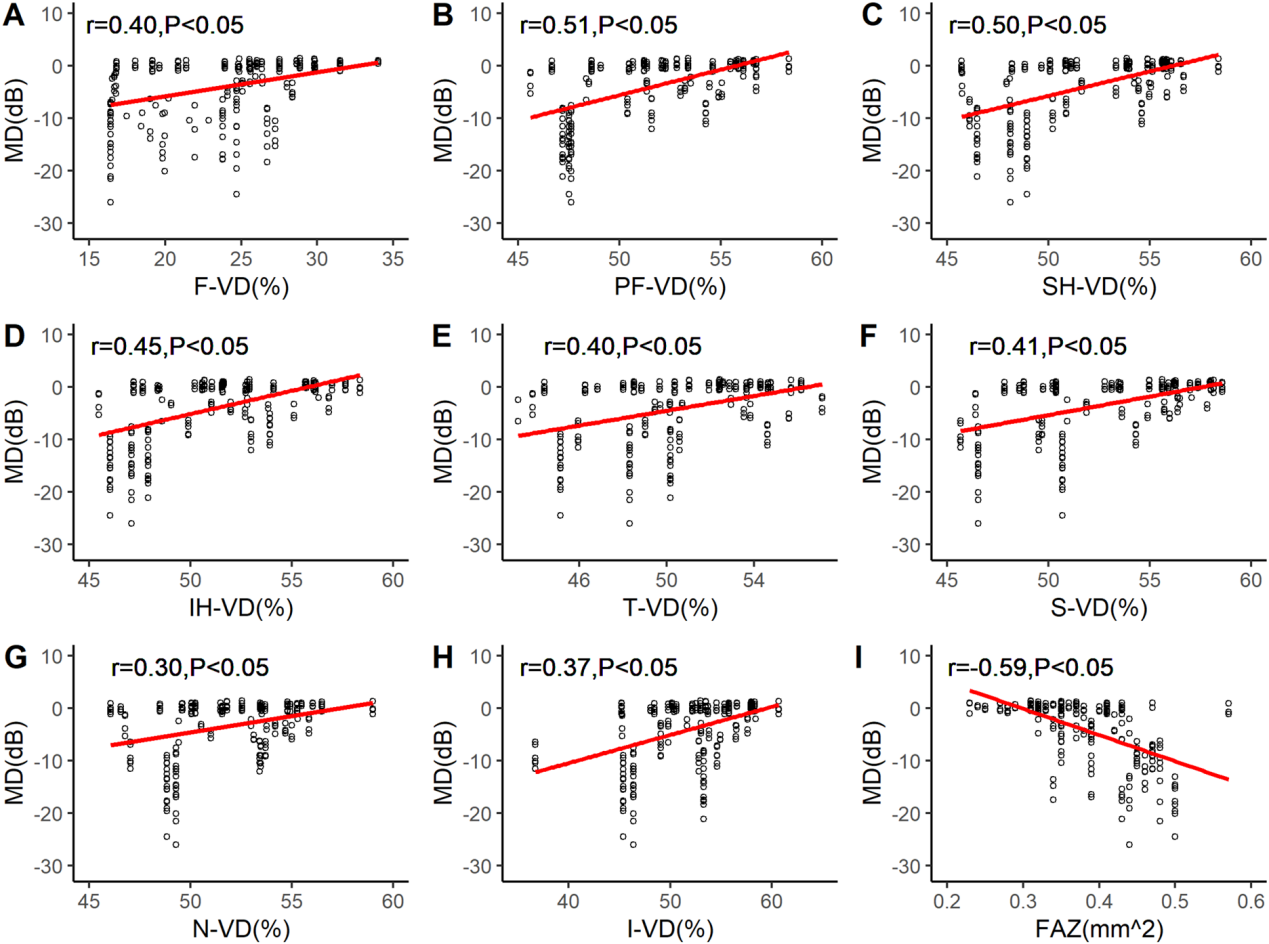
The scatter plots. **a**–**h** The correlation between VD in macula different areas and MD values. **i** The correlation between FAZ and MD values

All microcirculation parameters in macular area were correlated with MD values, and there were statistically significant differences ((*P* < 0.05). N-VD and I-VD were weakly correlated with MD values, while F-VD, PF-VD, SH-VD, IH-VD, T-VD, S-VD and FAZ were moderately correlated with MD values. The correlation between FAZ and MD value was the highest. The greater the FAZ value, the greater the visual field damage (Fig. 2).

MedCalc software was used to compare the diagnostic ability of VD and RNFLT for APACG. For the mild APACG versus PACS group, the diagnostic abilities of VD (AUC: 0.704–0.760) and RNFLT (AUC: 0.613–0.672) in the macular area were both low, and the difference in AUCs between the two groups was not statistically significant (*P* > 0.05). For the moderate APACG versus PACS groups, the diagnostic ability of VD (AUC: 0.902–0.942) in the macular area was high, and that of RNFLT (AUC: 0.805–0.842) was moderate. The AUCs of SH-VD, IH-VD, T-VD, S-VD, N-VD, and I-VD were statistically significantly greater than those of RNFLT in the corresponding area, which means that the diagnostic ability was greater (*P* < 0.05). For the severe versus the PACS groups, the diagnostic abilities of VD (AUC: 0.900–0.958) and RNFLT (AUC: 0.828–0.887) in the macular area were both improved, and the difference between the two AUCs were reduced. The AUCs of PF-VD, SH-VD, IH-VD, S-VD, and N-VD were statistically significantly greater than those of RNFLT in the corresponding area, which means that the diagnostic ability was greater (*P* < 0.05). With APACG exacerbation, the diagnostic abilities of F-VD and RNFLT gradually improved. The diagnostic sensitivity of VD in the macular area for distinguishing moderate and severe APACG was higher than that of the structural parameter RNFLT (Table 3).

**Table 3:** Comparison between VD and RNFLT in the diagnostic ability for APACG

Variable	Mild versus PACS			Moderate versus PACS			Severe versus PACS		
	AUC	Difference value	*P*	AUC	Difference value	*P*	AUC	Difference value	*P*
F-VD (%)	0.729 (0.649–0.800)	0.116	0.09	0.902 (0.843–0.945)	0.0765	0.10	0.900 (0.839–0.944)	0.0645	0.15
F-RNFLT (µm)	0.613 (0.528–0.693)			0.826 (0.755–0.883)			0.836 (0.764–0.893)		
PF-VD (%)	0.726 (0.645–0.797)	0.0904	0.16	0.915 (0.858–0.954)	0.0728	0.09	0.933 (0.879–0.968)	0.0956	0.02*
PF-RNFLT (µm)	0.636 (0.551–0.715)			0.842 (0.774–0.896)			0.837 (0.766–0.894)		
SH-VD (%)	0.746 (0.667–0.815)	0.0741	0.31	0.930 (0.876–0.965)	0.125	≤ 0.01*	0.948 (0.897–0.978)	0.119	≤ 0.01*
SH-RNFLT (µm)	0.672 (0.589–0.748)			0.805 (0.732–0.865)			0.828 (0.756–0.887)		
IH-VD (%)	0.708 (0.627–0.781)	0.0648	0.29	0.933 (0.881–0.968)	0.0973	≤ 0.01*	0.947 (0.896–0.977)	0.104	0.03*
IH-RNFLT (µm)	0.644 (0.559–0.722)			0.836 (0.767–0.891)			0.842 (0.772–0.898)		
T-VD (%)	0.709 (0.628–0.782)	0.0784	0.19	0.942 (0.891–0.973)	0.110	≤ 0.01*	0.920 (0.862–0.959)	0.0770	0.06
T-RNFLT (µm)	0.631 (0.546–0.710)			0.831 (0.761–0.887)			0.843 (0.772–0.899)		
S-VD (%)	0.760 (0.682–0.828)	0.111	0.10	0.921 (0.866–0.959)	0.106	≤ 0.01*	0.958 (0.911–0.985)	0.0933	0.04*
S-RNFLT (µm)	0.649 (0.565–0.727)			0.816 (0.744–0.874)			0.865 (0.797–0.916)		
N-VD (%)	0.742 (0.662–0.811)	0.0994	0.16	0.912 (0.855–0.952)	0.0912	0.03*	0.944 (0.893–0.976)	0.0802	0.04*
N-RNFLT (µm)	0.642 (0.558–0.721)			0.821 (0.750–0.878)			0.864 (0.797–0.916)		
I-VD (%)	0.704 (0.622–0.777)	0.0528	0.47	0.917 (0.860–0.955)	0.0934	0.04*	0.938 (0.885–0.972)	0.0512	0.25
I-RNFLT (µm)	0.651 (0.567–0.729)			0.823 (0.752–0.881)			0.887 (0.823–0.934)		

DeLong test: the value represents AUCs within 95% confidence interval

*APACG* acute primary angle closure glaucoma; *PACS* primary angle closure suspect; *VD* vessel density; *RNFLT* retinal nerve fiber layer thickness; *F* fovea; *PF* parafovea; *SH* superior hemiretina; *IH* inferior hemiretina; *T* temporal; *S* superior; *N* nasal; *I* inferior

*P < 0.05, showing that the difference was statistically significant

**Fig. 3:**
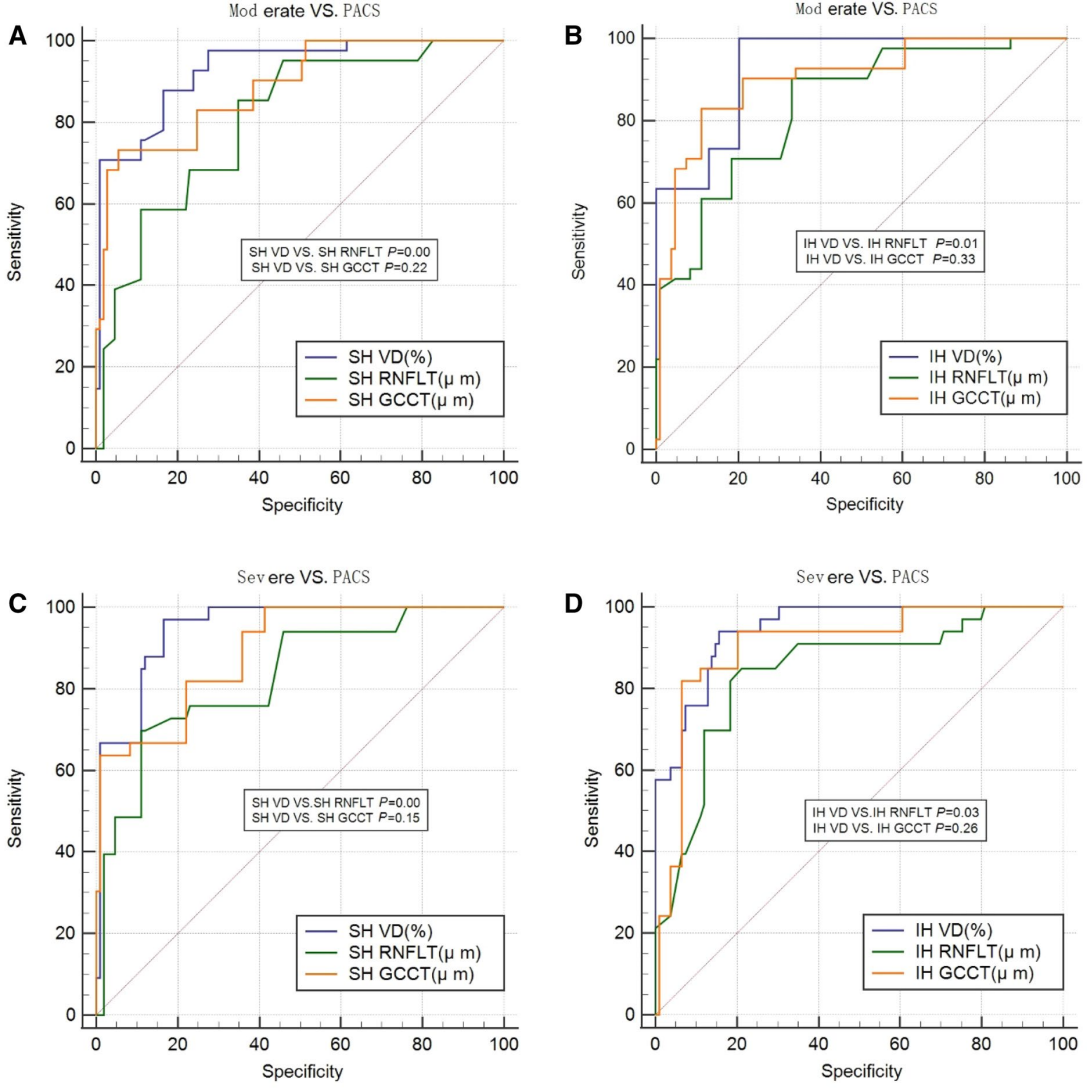
Receiver operating characteristic (ROC) curve. In the moderate versus PACS groups, **a** comparison of ROC curves between SH VD and RNFLT and GCCT; **b** comparison of ROC curves between IH VD and RNFLT and GCCT. In the severe versus PACS groups, **c** comparison of ROC curves between SH VD and RNFLT and GCCT. **d** Comparison of ROC curves between IH VD and RNFLT and GCCT

MedCalc software was used to compare the diagnostic abilities of VD and GCCT for APACG. For the mild APACG versus PACS groups, the AUC values of SH-VD and IH-VD were larger than those of GCCT in the corresponding area, but the diagnostic abilities of VD and GCCT were both low, and the difference was not statistically significant (*P* > 0.05). For the moderate APACG versus PACS and severe APACG versus PACS groups, the diagnostic abilities of VD and GCCT improved, and the AUC values of SH-VD and IH-VD remained higher than those of GCCT in the corresponding area. Nevertheless, the difference was not statistically significant (*P* > 0.05; Table 4, Fig. 3).

**Table 4:** Comparison between VD and GCCT in the diagnostic ability for APACG

	Mild APACG versus PACS			Moderate APACG versus PACS			Severe APACG versus PACS		
	AUC	Difference value	*P*	AUC	Difference value	*P*	AUC	Difference value	*P*
SH-VD (%)	0.746 (0.667–0.815)	0.0932	0.16	0.930 (0.876–0.965)	0.0433	0.22	0.948 (0.897–0.978)	0.0550	0.15
SH-GCCT (µm)	0.653 (0.569–0.731)			0.887 (0.825–0.932)			0.893 (0.830–0.938)		
IH-VD (%)	0.708 (0.627–0.781)	0.0637	0.32	0.933 (0.881–0.968)	0.0318	0.33	0.947 (0.896–0.977)	0.0409	0.26
IH-GCCT (µm)	0.645 (0.560–0.723)			0.902 (0.842–0.944)			0.906 (0.845–0.948)		

DeLong test: the value represents AUCs within 95% confidence interval

*APACG* acute primary angle closure glaucoma; *PACS* primary angle closure suspect; *VD* vessel density; *GCCT* ganglion cell complex thickness; *MD* mean deviation; *SH* superior hemiretina; *IH* inferior hemiretina

## Discussion

In recent years, increasing attention has been paid to the role of abnormal intraocular blood flow or vascular regulation ability in the development and progression of various types of glaucoma. OCTA technology has mainly been used to study POAG, and the difference between POAG and primary angle closure glaucoma to date. The widespread use of OCTA technology in clinics has enhanced our understanding of the retinal microcirculation state in APACG patients with normal IOP after an acute attack. VD and structural lesions of the affected eyes continue to develop after an APAC attack [30] due to secondary degenerative injuries [31]. In the present study, we focused on evaluating the vascular structure of the superficial retina, in the macula, by using OCTA technology to quantify microcirculatory function, and evaluated the diagnostic ability of this approach for identifying APACG, compared to traditional retinal structural parameters. We found that damage to the VD and FAZ in the macula increased with APACG severity. VD in the macular superficial layer showed a higher diagnostic ability than RNFLT, which was equivalent to that of GCCT.

In the present study, no statistically significant difference in IOP was found among the five groups on OCTA examination, indicating that, other than IOP, the main influencing factor, the VD of each macular area decreased gradually and the FAZ increased gradually with APACG severity. Compared to the healthy control and PACS groups, the mild APACG group had a lower F-VD and larger FAZ, suggesting that these two parameters can be used as sensitive indicators of retinal microcirculation damage in patients with APACG. In the moderate APACG vs. PACS group, the diagnostic ability of VD was higher than that of the RNFLT in six macular regions, while in the severe APACG vs. PACS group, the diagnostic ability of VD was higher than that of the RNFLT in five macular regions. The diagnostic abilities of VD and the GCCT in each macular partition were similar in the three APACG groups. These results indicated that, compared with RNFLT, which is traditionally used as the structural indicator, VD had a higher diagnostic value for APACG, which was comparable to that of the GCCT, a classic indicator of glaucoma damage.

Previous studies have found that the VD around the optic nerve head after an APAC was still low in cases with normal IOP, which was similar to the first conclusion in this study: VD in each macular sector after the acute attack was still low in cases with normal IOP, and decreased with increasing APACG severity [32]. Due to the limitations of the examination methods, APACG is usually characterized by anterior segment ischemia, and 52.5% of patients with APACG had changes in iris ischemia [33]. It has been believed that the iris stroma is least robust against damage caused by IOP elevation during an APAC. By using OCTA, it was seen that the damage to the retina caused by transient IOP elevation was also significant. The decrease in retinal VD leads to chronic damage, due to ischemia and hypoxia. Of note, the VD as assessed by OCTA was not an estimate of the blood flow velocity. Instead, it should be understood as changes, such as shedding of capillaries or slowing of blood flow velocity in vessels. Therefore, in addition to the application of IOP-lowering drugs, drugs used for improving microcirculation should also be considered as adjuvant therapy in daily clinical treatment, which may alleviate the subsequent effects of acute attack on retinal microcirculation.

Previous studies on POAG found that the retinal capillary density in a normal population was different from that in patients with moderate POAG, and that there was no difference between moderate and severe POAG patients [34]. This was consistent with the VD change trend in the macular area for different degrees of APACG in this study. Compared to the healthy control and PACS groups, VD in all areas of the macula of the moderate and severe APACG groups were significantly decreased. However, in the comparison between the moderate and severe APACG groups, there was no significant difference in VD in three macular sectors. This might be because early glaucoma is characterized by progressive microvascular changes, and as the disease progresses to a severe stage, microvascular changes were reduced with the floor effect caused by the thinning of the retinal nerve fiber layer and RGC apoptosis. Therefore, OCTA parameters were better able to diagnose the disease early rather than late [35].

The FAZ progressively increased under conditions of advancing age, diabetic retinopathy, retinal vein obstruction, and so forth [36–38]. This was because macular capillary loss, a characteristic of ischemic and vascular occlusive retinal disease, led to significant changes in the FAZ, as assessed by the area, perimeter, etc., of the FAZ [39–41]. The association between the FAZ and glaucoma is not well understood. Studies have shown that patients with glaucoma with central VF defects had larger and irregularly-shaped FAZs than those with normal VFs or peripheral VF defects [42, 43]. In addition, patients with NTG had a larger FAZ area and decreased peripheral vascular density. OCTA technology was used to demonstrate that NTG is an ischemic disease, characterized by vascular dysfunction [44]. In the present study, the FAZ changed significantly in the early stage of APACG, indicating that it is a sensitive indicator of APACG-induced changes in retinal circulation.

Prior studies have shown that VD as measured by OCTA could be used to distinguish healthy eyes from glaucomatous eyes, as well as to distinguish between healthy people and those with suspected glaucoma [45, 46]. Henry et al. studied the AUCs of glaucomatous and healthy eyes. They showed that the RNFLT (0.95) and GCCT (0.95) had the highest AUCs, followed by macular VD (0.94) and peripapillary VD (0.93) [47], which indicated that the diagnostic ability of VD in the macular area was similar to that of other known indicators, such as RNFLT and GCCT, which was consistent with the results of the present study. In addition, it has been suggested that changes in the peripapillary VD and macular VD as obtained by OCTA may be a new method for glaucoma staging [48]. The advantages of using macular VD for the diagnosis and assessment of APACG are as follows: First, the damage of early glaucoma might occur more clearly on the omentum, away from the optic nerve head [49]; therefore, early changes in peripapillary VD or RNFLT may not be detected. Second, some refractive errors, particularly astigmatism, are typically characterized by a slanted appearance and atrophy of the temporal optic nerve head, and perfusion of the retina of the optic nerve head is less than that of emmetropic eyes, which could impede the differential diagnosis of APACG and normal eyes [50, 51].

This study had some limitations. The scan pattern of the 6 × 6 mm^2^ region in the macular area had greater diagnostic accuracy than that of the 3 × 3 mm^2^ region, because a smaller scan range might miss part of the GCCT area [34]. However, if APACG subjects are examined with pupil dilation, the risk of APAC attacks is increased. Therefore, in this study, it was more reasonable to select the 3 × 3 mm^2^ region in subjects with undilated pupils. Another potential effect was that IOP-lowering eye drops may have altered eyeball hemodynamics and retinal vascular autoregulation. For example, carbonic anhydrase inhibitors and prostaglandin analogs could result in vessel dilatation and increased blood flow [52, 53]; this effect persists 1–4 weeks after discontinuation. Therefore, these medications were not discontinued during the examination. Several studies have found that a decrease in the VD of the optic nerve head and the corresponding macular area could be detected in patients with glaucoma with unilateral VF defects [54]. The VD (59.0 and 51.1%) in the hemi-optic nerve head and macular areas for the intact VF were higher than those for the affected VF (54.7 and 48.3%, respectively), but was lower than that of healthy eyes (62.4 and 53.8%, respectively). These findings suggest that vascular changes may also occur before VF defects are detectable. Therefore, VF parameters should be added for comparison, which may facilitate an understanding of the correlation between VD and structure and function in patients with APACG.

In conclusion, for cases of APACG with IOP that returns to normal after treatment, the VD was lower and the FAZ was smaller in the macular superficial layer than in healthy eyes, and the damage to the VD and FAZ was aggravated with APACG severity. VD in the macular superficial layer showed a higher diagnostic ability than RNFLT, and was equivalent to that of GCCT for detecting APACG. Further research is needed to establish whether a similar conclusions can be reached in a larger APACG population and in more types of glaucoma, and to understand the role of VD in the pathophysiological mechanism of glaucoma.

## Data Availability

The data used to support the findings of this study are available from the corresponding author upon request.
